# Green approach for tracking the photofate of ciprofloxacin and levofloxacin in different matrices adopting synchronous fluorescence spectroscopy: a kinetic study

**DOI:** 10.1098/rsos.221086

**Published:** 2023-01-18

**Authors:** Mohammed E. Draz, Dalia El Wasseef, Nahed El Enany, Mary E. K. Wahba

**Affiliations:** ^1^ Department of Pharmaceutical Chemistry, Faculty of Pharmacy, Delta University for Science and Technology, Gamasa 11152, Egypt; ^2^ Department of Medicinal Chemistry, Mansoura University, Mansoura 35516, Egypt; ^3^ Department of Pharmaceutical Analytical Chemistry, Faculty of Pharmacy, Mansoura University, Mansoura 35516, Egypt; ^4^ Department of Pharmaceutical Chemistry, Faculty of Pharmacy, New Mansoura University, P.O. Box 7723730, New Mansoura, Egypt

**Keywords:** ciprofloxacin, levofloxacin, synchronous fluorescence spectroscopy, photolysis, degradation kinetics, greenness

## Abstract

First derivative synchronous fluorescence spectroscopy (FDSFS) was applied to detect and quantify either ciprofloxacin (CIP) or levofloxacin (LEV) simultaneously with their photodegradation products, where the photolytic pathway for each analyte was found to be pH dependent. Under the guidance of early published articles, the structure of the produced photolytic products could be concluded, and further related to their resultant fluorescence spectra. The proposed method was subjected to full validation procedure which enables its application in investigating the photodegradation kinetics for both drugs. The obtained kinetic parameters were in accordance with previous reports and could be linked to predict the antibacterial activity of the resultant photodegradation products. These facts prove the suitability of the suggested FDSFS to serve as a stability-indicating assay method and to trace the photofate of CIP and LEV in the ecosystem as potential contaminants. Furthermore, the greenness of the suggested analytical methodology was evaluated via ‘Green Analytical Procedure Index’ (GAPI), which classifies it as an eco-friendly assay. Eventually, no extraction, treatment or preparation steps were needed during all analysis steps, which renders the proposed assay an appealing tool in environmental analysis.

## 1. Introduction

When the well is dry, we know the worth of water, a historical saying by Benjamin Franklin which encourages the protection of water resources, carrying a prospective insight into human behaviour toward nature. During the twentieth century, a significant six-fold inflation in water consumption has been recorded [1–3], besides, a crucial misuse of different water resources via contamination linked to various human activities has turned out to release questionable health issues [1,4].

Antibiotic resistance is considered an emergent concern that negatively affects both public health and environmental safety [5]. One of the major routes responsible for its advent is the proliferation of antibiotics (AB) from the wastewater treatment plants (WTP) which receive wastewater contaminated with AB from many sources, i.e. pharmaceutical companies, hospitals, households and others [1,6,7]. Meanwhile, WTP cannot eliminate AB, hence they propagate to drinking, surface and groundwater, in addition to WTP effluents [1,6,7].

Fluoroquinolone antibiotics (FQN) are considered one of the AB classes that are extensively used by humans and in veterinary medicine owing to their broad antibacterial spectrum [7–9]. They are subjected to partial metabolism, being excreted in their pharmacologically active intact form [7–9]. Besides combating treatment in WTP, FQN are highly stable to microbial degradation, hydrolysis and high temperature [7,10,11], which explains their classification as the most abundant class of antibiotics in surface water [7,12–14]. These facts accentuate the need for the presence of an alternative treatment for the efficient removal of these pharmaceuticals, where their presence in the environment even in minute amounts accounts for the development of resistance [1,7].

The fate of pharmaceutical contaminants in the ecosystem is determined by biotic and abiotic processes [7], where hydrolysis and photodegradation are the major pathways for abiotic providence in surface water [7]. Pharmaceuticals resistant to hydrolysis like FQN are subjected to direct and indirect photolysis as the principal abiotic degradation route [7,15]. Although self-sensitized photolysis, a major indirect photolysis process applying reactive oxygen-containing species like OH**^·^** radicle, has been emphasized for FQN in previous reports [7,16,17], it is not expected to be prevalent in the ecological system where it necessitates the presence of initial high concentrations of contaminants, which is not expected in environmental matrices [7,16,17]. Thus direct photodegradation represents a significant route to eliminate FQN from surface water [7]. As documented in previous reports, only photodegradative destruction of the main core of the parent FQN drug inhibits its antibacterial activity and consequently subsides the development of resistance [18–20].

Decarboxylation, defluorination and quinolone ring hydrolysis are the main photolytic pathways resulting in FQN core destruction [18–20]. On the other hand, some of the intermediates like the N-4 de-alkylated derivatives—which are formed during the primary photodegradation process—possess almost the same antibacterial activity as the parent drug [18–20], which implies that their accumulation in the aquatic environment is not desirable. The photofate of different FQN members is influenced by many variables: the pH, which is considered a major factor, and the frequently encountered components in the ecosystem, i.e. organic matter, Cl^−^, ${\mathrm{NO}}_{3}^{-}$ and ${\mathrm{SO}}_{4}^{2-}$ [7,16,21].

**Figure 1.**
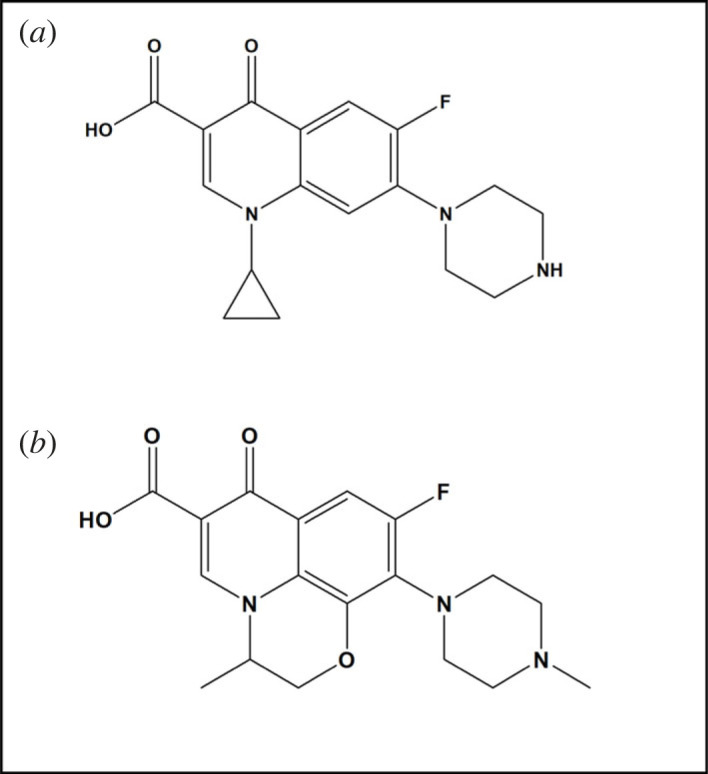
Structural formulae of (*a*) CIP and (*b*) LEV.

Ciprofloxacin (CIP), 1-cyclopropyl-6-fluoro-1,4-dihydro-4-oxo-7-(1-piperazinyl)-3-quinolinecarboxylic acid [22] (figure 1*a*), and levofloxacin (LEV), (−)-(S)-9-fluoro-2,3-dihydro-3-methyl-10-(4-methyl-1-piper-azinyl)-7-oxo-7H-pyrido[1,2,3-de]-1,4-benzoxazine-6-carboxylic acid [22] (figure 1*b*), are two members of the FQN family that are extensively used in veterinary medicine both to treat and to promote animal growth, where their release in surface water is associated with animal waste [1,23].

Wastewater treatment via photocatalysis for the efficient removal of CIP and LEV was a subject of concern in many articles, whereas the review articles presented by Shurbaji *et al.* [1] and Saya *et al.* [24] represent a good deal to these researches. However, analytical methods proposed for simultaneous estimation of either drug with its photodegradation products are limited, and usually employ high-performance liquid chromatography coupled with mass spectrometry (HPLC/MS) [25–28]. Even though it has remarkable advantages, many limitations hinder the use of HPLC/MS widely: it requires experienced trained analysts for its operation, has a high cost in terms of analysis and maintenance, only employs volatile buffers and consumes large volumes of organic solvents contradicting basic principles of green chemistry [29]. Therefore, there is an urge to develop an alternative analytical method with comparable sensitivity and selectively to HPLC/MS, which motivated the authors to carry out this investigation.

The availability of spectrofluorometer in most analytical laboratories, its ease of operation and its affordable cost encourage researchers—especially those located in developing countries—to employ it for the analysis of environmental samples, which renders the proposed method a suitable candidate for this purpose. In this work, photodegradation of CIP and LEV was carried out at the specified pH, and then simultaneous determination of each analyte with its photodegradation products was performed applying first derivative synchronous fluorescence spectroscopy (FDSFS).

Constant wavelength synchronous fluorescence spectroscopy (CWSFS) was the approach adopted in this work owing to its unique properties, being highly selective, providing well-resolved peaks in multi-component mixtures, simplifying complex spectra and producing sharp bands with narrow width [30]. Combination of CWSFS with derivative technique results in a synergistic effect on both selectivity and sensitivity [30], hence it was embraced in the present work. Additionally, the suggested method was subjected to greenness assessment where it proved to be green according to ‘Green Analytical Procedure Index’ (GAPI) proposed by Płotka-Wasylka [31].

## 2. Experimental

### 2.1. Apparatus

Shimadzu spectrofluorophotometer RF-6000 was used during all relative fluorescence intensity measurements (serial no. A402458, Shimadzu Corporation, Kyoto, Japan).

Jenway 3520 pH/temp meter, combined with glass pH electrode was used for pH adjustment (Jenway, Staffordshire, United Kingdom).

### 2.2. Materials and reagents

Ciprofloxacin (CIP) was kindly provided by Eipico Pharmaceutical Company, Tenth of Ramadan city, Egypt. Levofloxacin (LEV) was kindly supplied by Memphis Pharmaceuticals and Chemical Industries Co. S.A.E., Cairo, Egypt.

Methanol (HPLC grade), was purchased from TEDIA Way, Fairfield, United States. Hydrochloric acid and sodium hydroxide were purchased from EL Nasr company for intermediate chemicals, Giza, Egypt.

### 2.3. Stock and working standard solutions

Ciprofloxacin and levofloxacin stock standard solutions were prepared at a concentration of 1.0 mg ml^−1^ by dissolving 100.0 mg of each analyte in the minimum amount of methanol, 5 ml, and completing the volume to 100.0 ml in a volumetric flask with deionized water obtained from our laboratory. Working standard solutions for each pharmaceutical were prepared by further dilution with deionized water.

### 2.4. Preparation of pharmaceutical wastewater

Pharmaceutical wastewater (PWW) was prepared to resemble the composition of wastewater resulting from pharmaceutical companies. Based on a previous article [7], it was prepared in tap water to contain 30 mg l^−1^ ascorbic acid, 50 mg l^−1^ citric acid, 100 mg l^−1^ saccharose, 230 mg l^−1^ disodium hydrogen phosphate and 1 gm l^−1^ sodium chloride, adjusting the pH to 7 by 0.1 M HCl and 0.1 M NaOH.

### 2.5. Construction of calibration graphs

Different concentrations covering the linearity ranges of 1.0–15.0 and 1.0–20.0 µg ml^−1^ for CIP and LEV respectively were prepared by transferring appropriate volumes of the stock solutions to 10.0 ml volumetric flasks, and completing the volume to the mark using deionized water as a diluent. By fixing Δ*λ* at 100 nm, SFS for different concentrations of either drug was carried out, where the scan speed was fixed at 6000 nm min^−1^ and the bandwidth of both excitation and emission monochromators was maintained at 5 nm. The measured synchronous fluorescence spectra were then manipulated by subjecting them to first derivatization using Shimadzu Lab Solution RF software. The amplitudes of the first derivative peaks of CIP were measured at *λ*_em_ of either 437 or 420.4 nm when simultaneously measured with its photolytic products obtained at pH 4 or (pH 8/PWW), respectively. On the other hand, LEV was measured at 460 nm when assayed with its photodegradation product obtained at pH 4. For both pharmaceuticals, the selected wavelengths of measurement represent zero-crossing points for the obtained photolytic products. After repeating each measurement in triplicate, and by plotting the amplitudes of the obtained first derivative peaks for each analyte—after subtraction from simultaneously performed blank experiments—versus the final concentration (µg ml^−1^), calibration graphs were obtained and regression equations were derived.

### 2.6. Photodegradation kinetics of CIP and LEV

Six working solutions (three for each drug) were prepared to reach a final concentration of 10.0 µg ml^−1^. Two solutions were prepared by spiking laboratory-prepared PWW with appropriate volumes of stock standards of either CIP or LEV. Through serial dilution of the stock solutions of either analytes using deionized water, another four solutions were obtained (two for CIP having a final pH value of 4 or 8, and another two corresponding solutions for LEV).

The prepared solutions were subjected to photodegradation via exposure to a UV lamp of 254 nm. Suitable volumes were withdrawn according to a scheduled timetable: every minute (during the first 10 min of photolysis), followed by withdrawal every 10 min (till one hour of photolysis), and eventually every 30 min (till 7 h of photolysis). The withdrawn solutions were then analysed according to the procedures mentioned under section ‘Construction of calibration graphs’.

## 3. Results and discussion

### 3.1. Selection of Δ*λ*

**Figure 2.**
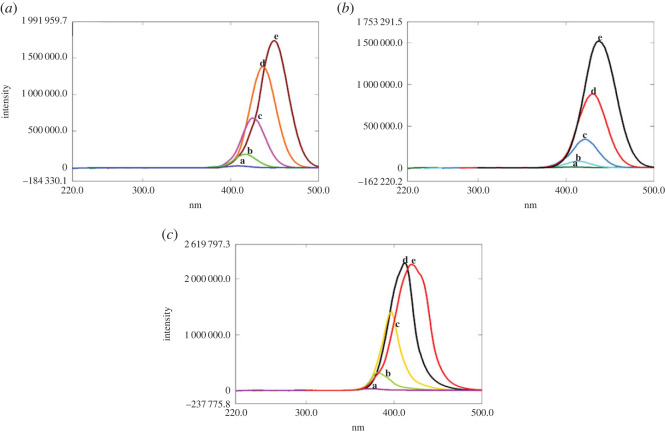
(*a*) Synchronous fluorescence spectroscopy of 10.0 µg ml^−1^ CIP at Δ*λ*: (a) 20 nm, (b) 40 nm, (c) 60 nm, (d) 80 nm and (e) 100 nm. (*b*) Synchronous fluorescence spectroscopy of the photolytic product of CIP (10.0 µg ml^−1^) at pH 4 at Δ*λ*: (a) 20 nm, (b) 40 nm, (c) 60 nm, (d) 80 nm and (e) 100 nm. (*c*) Synchronous fluorescence spectroscopy of the photolytic product of CIP (10.0 µg ml^−1^) at pH 8 at Δ*λ*: (a) 20 nm, (b) 40 nm, (c) 60 nm, (d) 80 nm and (e) 100 nm.

**Figure 3.**
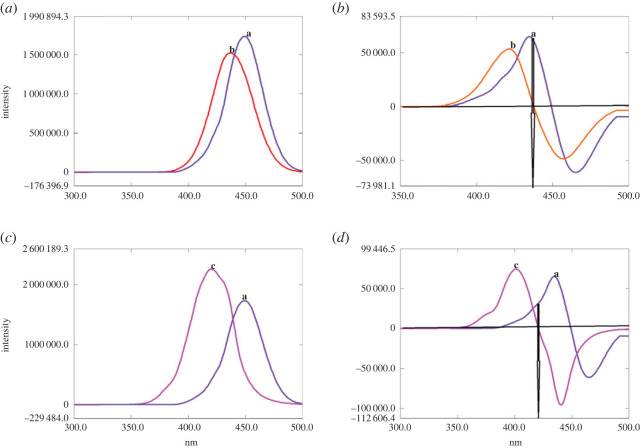
(*a*) Synchronous fluorescence spectroscopy of CIP (a) (10.0 µg ml^−1^) in presence of its photolytic product at pH 4 (b) at Δ*λ* = 100 nm. (*b*) First derivative synchronous fluorescence spectroscopy of CIP (a) (10.0 µg ml^−1^) in presence of its photolytic product at pH 4 (b) at Δ*λ* = 100 nm. (*c*) Synchronous fluorescence spectroscopy of CIP (a) (10.0 µg ml^−1^) in presence of its photolytic product at pH 8 (c) at Δ*λ* = 100 nm. (*d*) First derivative synchronous fluorescence spectroscopy of CIP (a) (10.0 µg ml^−1^) in presence of its photolytic product at pH 8 (c) at Δ*λ* = 100 nm.

In CWSFS, extensive studies for the selection of Δ*λ* should be performed owing to its significant influence on peak intensity and band width [30]. Figure 2*a–c* represents the SFS of CIP and its photodegradation products (at pH 4 or pH 8) at different Δ*λ* ranging from 20 to 100 nm. It was found that Δ*λ* = 100 nm was the optimum value, accomplishing the best resolution, and permitting quantification of the parent antibiotic at 437 or 420.4 nm after the first derivatization when simultaneously assayed with its photolytic products obtained in acidic or alkaline medium respectively (figure 3*a–d*).

**Figure 4.**
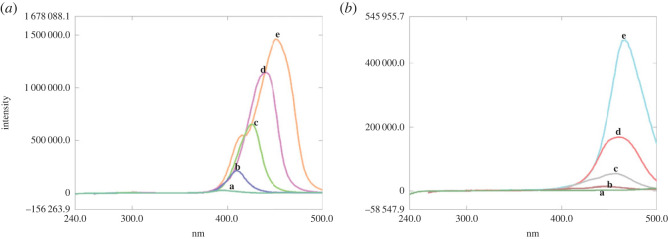
(*a*) Synchronous fluorescence spectroscopy of 10.0 µg ml^−1^ LEV at Δ*λ*: (a) 40 nm, (b) 60 nm, (c) 80 nm, (d) 100 nm and (e) 120 nm. (*b*) Synchronous fluorescence spectroscopy of the photolytic product of LEV (10.0 µg ml^−1^) at pH 4 at Δ*λ*: (a) 40 nm, (b) 60 nm, (c) 80 nm, (d) 100 nm and (e) 120 nm.

**Figure 5.**
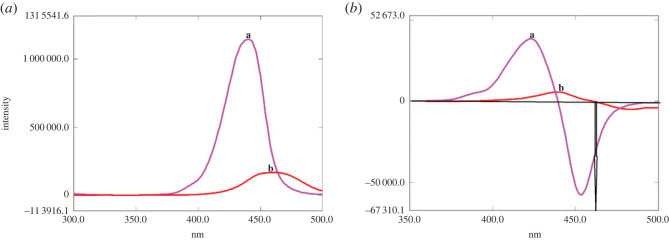
(*a*) Synchronous fluorescence spectroscopy of LEV (a) (10.0 µg ml^−1^) in presence of its photolytic product at pH 4 (b) at Δ*λ* = 100 nm. (*b*) First derivative synchronous fluorescence spectroscopy of LEV (a) (10.0 µg ml^−1^) in presence of its photolytic product at pH 4 (b) at Δ*λ* = 100 nm.

In the same manner, the SFS of LEV and its photodegradation product (obtained in acidic medium) were measured at Δ*λ* values ranging from 40 to 120 nm (figure 4*a,b*). After careful evaluation, Δ*λ* value of 100 nm was selected giving optimum separation, endorsing quantification of LEV at 460 nm after first derivatization without interference from its photolytic product (figure 5*a,b*).

### 3.2. Pathway of photodegradation of CIP and LEV

Since FQN are zwitterions, their photodegradation is significantly influenced by the pH, which affects both the reaction kinetics and the pathway of photolysis [7,32]. Regarding CIP, its photolytic pathways at both pH 4 and pH 8 were previously studied, and the resultant products were separated and identified by HPLC-MS/MS [7]. At pH 4, besides defluorination and rupture of the cyclopropane, cleavage of the piperazine ring followed by oxidation takes place [7]. While at pH 8 the photodegradation pathway was completely different, where at first cleavage of the bond between the nitrogen atom of the piperazine ring and the carbon atom (at position 7) of the quinolone ring arises, yielding (intermediate#1) carrying the 7-aminoquinolne structure. This was followed by deamination of (intermediate#1) accompanied by solvolysis of the fluorine atom to be substituted by a hydroxyl group (intermediate#2). The phenolic structure of (intermediate#2) renders it liable to oxidative degradation to finally yield a pyridone dicarboxylic acid derivative [7]. Scheme 1 demonstrates the structure of the final photolytic products of CIP at both pH values.

**Figure:**
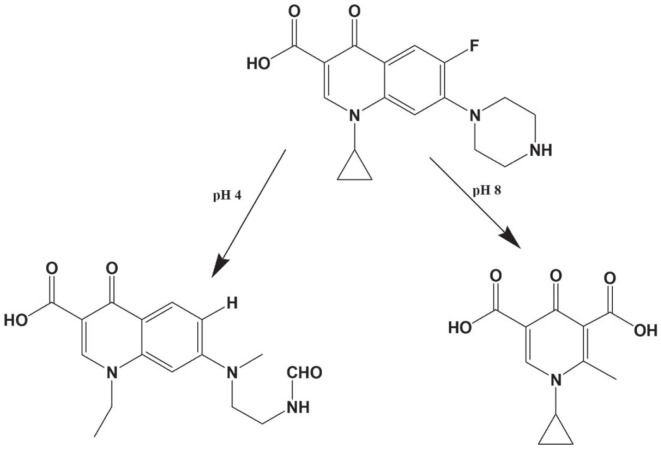
Scheme 1.

Cleavage of the piperazine ring at pH 4 (scheme 1) reduces CIP rigidity, and hence there is a subsequent decrease in the fluorescence intensity of the degradation product compared with the parent drug results (figure 3*a,b*). Moreover, the resultant product with its open ring structure is more energetic and hence its *λ*_em_ is lower than CIP (figure 3*a,b*).

On the other hand, at pH 8, conversion of benzenoid structure (in CIP) to quinoid structure (in its photolytic product) (scheme 1), significantly enhances the fluorescence intensity of the latter (figure 3*c,d*). This effect is accompanied by a reduction in *λ*_em_ of the produced photolytic product (figure 3*c,d*), where the repulsive forces between the adjacent carboxylate anions (scheme 1) impart instability to the formed product, where the anionic form of the formed photolytic product is expected to be prevalent in alkaline medium as indicated from the p*k*a values of CIP 6.1, 8.6 [33].

Regarding LEV, its photodegradation behaviour at pH 4 is quite similar to CIP, where it undergoes oxidative cleavage of the piperazine ring as documented by Lam & Mabury [34] (scheme 2), giving rise to a product that exhibits a remarkable decrease in its fluorescence (figure 5*a,b*). This influence on fluorescence intensity could be attributed to the loss of LEV rigidity—imparted by its fused ring structure—and augmented by repulsion between the closely spaced positively charged nitrogen atoms of the cleaved piperazine ring, which is exposed to take place at pH 4 by referring to LEV p*k*a values, 6, 8.1 [33].

**Figure:**
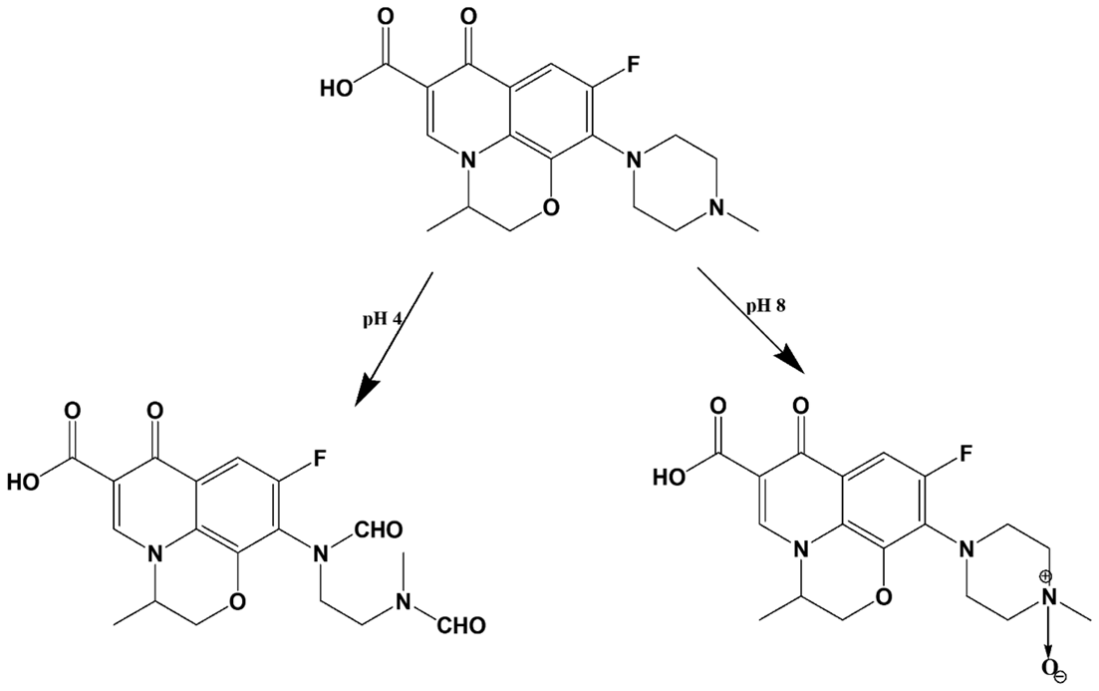
Scheme 2.

Meanwhile, LEV shows high stability to photolysis at pH 8, where its peak amplitude remains constant after irradiation for more than 7 hours. These findings are in accordance with those of Czyrski *et al.* [35] who reported only 5% degradation of LEV after 84 days of exposure to sunlight at this specified pH, where the resultant product was isolated and found to be the piperazine N-oxide derivative (scheme 2). These facts were formerly supported by Polishchuk *et al.* [32], who irradiated LEV at 254 nm for 2 hours at different pH values and traced the change in its luminescence spectra. At pH 8, no changes in the analyte spectrum were recorded over the entire irradiation period, which reinforces the experimental results obtained in this work.

### 3.3. Implication of CIP and LEV photodegradation kinetics on bactericidal activity

**Figure 6.**
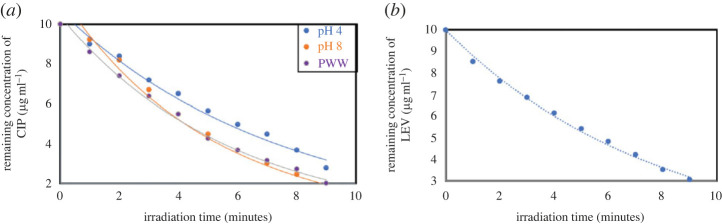
(*a*) Degradation profile of CIP (10.0 µg ml^−1^) at pH 4, pH 8, and in PWW. (*b*) Degradation profile of LEV (10.0 µg ml^−1^) at pH 4.

Complete photolysis of CIP (at pH 4, pH 8 and PWW) and LEV (at pH 4) was achieved in 10 min of irradiation following an exponential pattern (figure 6*a,b*), which is in accordance with previous reports [13]. Thus, for simultaneous determination of each analyte with its photolytic product (in each matrix), irradiation for not less than 10 min was applied to ensure complete photodegradation and formation of only one photodegradation product—without interference from intermediates formed during photodegradation pathways—hence preventing erroneous results during quantification. The initial concentration (of the parent antibiotics) used to perform the kinetic study (10.0 µg ml^−1^) was selected based on the fact that low concentrations result in higher reaction rates [7], hence the authors used a concentration simulating that encountered in ecosystems contaminated with FQN to yield representative realistic kinetic parameters. The short half-life times for the photolysis of both analytes, 3.5–5.6 min (table 1), demonstrate the significance of photodegradation in determining the photofate of such contaminants in the ecosystem.

**Table 1.** Kinetic parameters for the photolysis of CIP and LEV.

drug	matrix	regression equation	*K* (min^−1^)	*t*_1/2_ (min)	*R*^2^
CIP	pH 4	−0.133X + 1.385	0.133	5.2	0.9936
	pH 8	−0.196X + 1.984	0.196	3.5	0.9987
	PWW	−0.165X + 1.791	0.165	4.2	0.9971
LEV	pH 4	−0.124X + 1.261	0.124	5.6	0.9931

**Figure 7.**
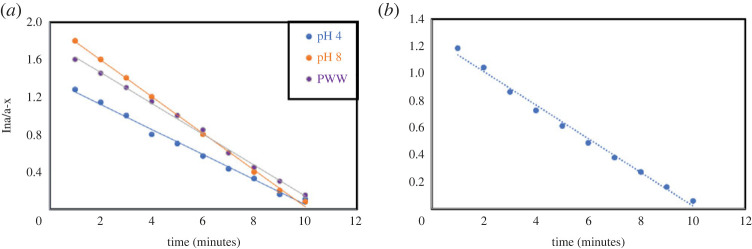
(*a*) First order plot for the photodegradation of CIP (10.0 µg ml^−1^) at pH 4, pH 8 and in PWW. (*b*) First order plot for the photodegradation of LEV (10.0 µg ml^−1^) at pH 4.

The photodegradation processes follow first-order kinetics as reflected from the linear relationship obtained between (ln a/a-x) versus irradiation time [36] (figure 7*a,b*), where (a) is the initial concentration of the intact drug (10.0 µg ml^−1^), and (a-x) is the remaining concentration of the analyte after irradiation at a time (t) in minutes [36]. By reviewing the obtained kinetic parameters for CIP photodegradation (table 1), it was found that the reaction rate constant (*K*) increases by pH elevation, which is formerly documented [7] expecting that the resultant anionic species at alkaline pH values promote photolysis yielding higher reaction rates than those produced from cations obtained in acidic media.

The reaction rate of CIP photolysis in synthetic PWW was lower than that at pH 8 (table 1), which could be ascribed to different components expected to be encountered in PWW. Both ascorbic acid and chloride ions are documented to suppress photodegradation [7,16] taking into consideration that the role of Cl^−^ is limited when compared with ascorbic acid, which is reported to absorb radiation competing with CIP, thus inhibiting its photolysis [7,16]. On the contrary, phosphate ions are recorded to promote photolysis [7,16]. Hence, the overall effect on the photodegradation rate of CIP in PWW results from the balance between contradictory contributions from different constituents [7,16].

Regarding LEV, its reaction rate constant (*K*) at pH 4 was lower than that of CIP (table 1), while at both pH 8 and in PWW, no change in peaks amplitudes was observed, as mentioned before, indicating its resistance to photolysis. This behaviour could be explained by referring to its chemical structure (figure 1*b*) carrying the benzoxazine fused ring moiety which imparts to LEV a remarkable inhibition of photoreactivity [37,38]. Despite being an advantage during dispensing LEV in various pharmaceutical formulations, its stability to photodegradation becomes an obstacle regarding its fate in the ecosystem. The produced photolytic product at pH 4 (scheme 2) presented by Lam & Mabury [34], still carries the intact structure of the quinolone ring, the carboxylic group, the mediator for DNA gyrase binding [19,39] and the fluorine atom which is reported to be unsusceptible to dehalogenation by irradiation [19]. These structural features suggest that the produced product still offers bactericidal activity although being lower than that of the parent antibiotic owing to piperazine ring cleavage [18–20]. Besides, at pH 8 and in PWW, LEV exists in its intact form for a considerably long time till its photolysis begins [35], forming eventually the piperazine N-oxide derivative [35] (scheme 2). The formed product carries all the structural criteria of the acidic photolytic product described formerly, in addition to keeping the piperazine ring intact [35], which confirms that its bactericidal activity is comparable to LEV [18–20]. All these facts warn of the emergence of resistance to LEV, classifying it as a critical environmental pollutant.

On the other hand, the obtained kinetic parameters (table 1) indicate that CIP is more susceptible to photolysis than LEV, where its photodegradation proceeds faster in slightly alkaline and neutral media than in acidic matrix. At pH 8, the obtained product is devoid of the quinolone ring structure, the fluorine atom and the piperazine ring [7] (scheme 1), predicting the loss of the antibacterial effect [18–20]. Meanwhile, the acidic photolysis product lacks both the fluorine atom and the piperazine ring, but still preserves the quinolone moiety [7] (scheme 1), indicating a crucial inhibition in bactericidal activity [18–20]. These findings support the liability of CIP to photolysis forming photolytic products devoid of any bactericidal effect, and launching an influential promising green pathway for its decay in the ecosystem.

Eventually, the obtained photolysis kinetic data (table 1) are comparable to those procured from early reports [7,16,40], proving the suitability of the suggested method in predicting reaction rate kinetic parameters.

### 3.4. Analytical method validation

A full validation study was performed according to the US Pharmacopeia (USP) [22] to establish a high assurance degree that the suggested analytical method can provide consistent results reflecting the quality of the tested analytes. Before undertaking validation, the full analytical system was checked to ensure that it is designed, calibrated and maintained adequately.

Linearity ranges were assessed by tracing the proportionality between the FDSFS peak amplitudes and the final concentration of each of CIP and LEV using the described experimental conditions, where the linearity ranges were found to be 1.0–15.0 and 1.0–20.0 µg ml^−1^, respectively.

Through the application of the suggested analytical method to the analytes, and comparing the obtained results with those of a well-characterized assay, the accuracy is evaluated by judging the nearness of the results of both methods [22]. Different concentrations of CIP and LEV were assayed by the proposed method over their linearity ranges, and the obtained data were then compared with those of the reported methods [41,42]. The adequate closeness between both methods was recoded as implemented from the Students' *t* test and variance ratio *F* test [43], in addition to the resultant high recovery percentages (table 2) proving the accuracy of the proposed method.

**Table 2.** Analysis of CIP and LEV in their pure form applying the proposed method.

parameter	taken (µg ml^−1^)	found (µg ml^−1^)	% recovery	comparison methods [41,42], % found
CIP	1.0	0.984	98.40	99.34
	2.0	1.965	98.25	101.42
	5.0	5.124	102.48	100.67
	7.0	7.087	101.24	
	10.0	9.876	98.76	
	12.0	11.735	97.79	
	15.0	14.879	99.19	
mean ± s.d.			99.44 ± 1.74	100.48 ± 1.05
*t* test			0.36 (2.447)^a^	
*F* test			2.75 (19.33)^a^	
LEV	1.0	0.982	98.20	98.65
	5.0	4.894	97.88	99.37
	7.0	6.923	98.90	100.79
	10.0	10.043	100.43	
	12.0	11.945	99.54	
	15.0	15.153	101.02	
	20.0	19.882	99.41	
mean ± s.d.			99.34 ± 1.13	98.33 ± 1.09
*t* test			0.51 (2.447)^a^	
*F* test			1.07 (19.33)^a^	

^a^Tabulated *t* and *F* values at *p* = 0.05 [43]. Each value represents the mean of three separate measurements.

The sensitivity of the proposed method was evaluated by estimating both limit of detection (LOD) and limit of quantification (LOQ) as stated by the USP [22] to be the analyte concentration yielding a peak: noise ratio of 3 : 1 (LOD) or 10 : 1 (LOQ). The LOD and LOQ values of CIP were 0.54 and 0.82 µg ml^−1^, with corresponding values of 0.49 and 0.73 µg ml^−1^ for LEV.

The assay procedure was applied on multiple homogeneous samples, measuring the agreement between the obtained data accounts for precision [22]. Reproducibility and repeatability are two measures of precision [22] assessed in this work. Precision, regarding both measures, is expressed in terms of standard deviation (s.d.), which demonstrates with its small values (table 3), satisfactory reproducibility and repeatability. Ruggedness, one of the reproducibility key elements, was estimated and expressed as variation within the laboratory [22]; i.e. carrying out the procedure on different days by the same analyst. Meanwhile, repeatability was evaluated through performing the proposed assay over a short time (same day), by the same analyst, using the same equipment [22].

**Table 3.** Precision data of the proposed method.

parameter	taken (µg ml^−1^)		% recovery	
	CIP	LEV	CIP	LEV
repeatability	1.0	1.0	99.32	98.21
	8.0	10.0	98.84	99.04
	15.0	20.0	99.95	99.37
mean			**99**.**37**	**98**.**87**
s.d.			**0**.**56**	**0**.**59**
ruggedness	1.0	1.0	98.23	99.31
	8.0	10.0	99.87	98.85
	15.0	20.0	100.04	100.23
mean			**99**.**38**	**99**.**46**
s.d.			**0**.**99**	**0**.**71**

The establishment of specificity was carried out by ensuring that quantification of the studied antibiotics was performed unambiguously in presence of their photolytic products [22], which was successfully accomplished (figures 3 and 5) owing to the unique features of CWSFS supplemented with derivatization as formerly described.

Evaluating the validation parameters of the proposed method with regard to the reference methods [41,42] reflected comparable accuracy and linearity, as reflected from recovery percentages and *R*^2^ values respectively (table 4). Meanwhile, the proposed method was superior to the reference methods offering wider linearity ranges for both drugs (table 4), permitting expansion of its application scope.

**Table 4.** Validation parameters for the assay of CIP and LEV by the proposed and reference methods.

parameter	proposed method		reference method for CIP [41]	reference method for LEV [42]
	CIP	LEV		
linearity range (µg ml^−1^)	1.0–15.0	1.0–20.0	0.5–10.0	0.36–2.88
LOD (µg ml^−1^)	0.54	0.49	0.11	0.36
LOQ (µg ml^−1^)	0.82	0.73	0.33	1.2
*R*^2^	0.9989	0.9995	0.9999	0.9983
% recovery	99.44	99.34	99.97	104.0

Sensitivity—expressed as LOD and LOQ—was found to be comparable in the case of analysing LEV by the proposed or the reference method [42] (table 4). On the other hand, LOD and LOQ for CIP using the reference method [41] (table 4) imply that it is more sensitive than the suggested method; however, the latter presents more reliable values. Ermer and Miller evaluated different approaches for calculating LOD and LOQ values in their book concerned with validation in pharmaceutical analysis [44]. They stated that relevant values for LOD and LOQ are provided only when degradation products and/or impurities are spiked to the concerned pharmaceutical, followed by application of the control test to retrieve the quantitative analysis data [44]. When spiking is not achievable, signal to noise approach is the only valid procedure to yield reliable values [44]. The proposed method followed the validation parameters stated by the USP [22] which adopted the aforementioned approach to evaluate sensitivity in terms of LOD and LOQ as previously described. On the other hand, the reference method for CIP [41] followed the International Council for Harmonisation guidelines [45], which is expected to result in unsubstantial sensitivity parameters since spiking was not carried out. These facts explain the accordance in sensitivity for LEV analysis by both the proposed and reference methods [42], which also followed signal to noise ratio approach to evaluate sensitivity.

### 3.5. Evaluation of the proposed method greenness applying the Green Analytical Procedure Index

**Figure 8.**
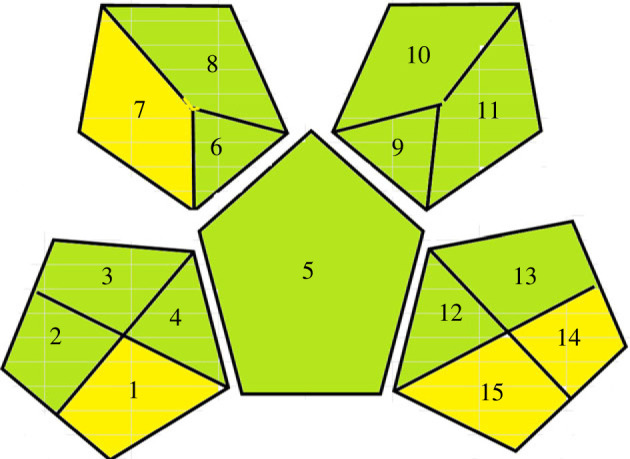
GAPI pictograms for greenness evaluation of the proposed method.

One of the crucial parameters that judge the analytical procedures' greenness is the time between sample collection and obtaining final analysis results [31]; i.e. multi-step analysis is expected to have a negative environmental impact [31]. Green Analytical Procedure Index (GAPI) is the most recent tool applied to evaluate the greenness of analytical methodologies [31], where it traces all the steps involved in assay procedures. This valuable tool utilizes pictograms to illustrate the obtained output [31], where each pictogram describes a specific category. Category 1 (steps 1–4) is concerned with sample collection, preservation, storage and transport, while category 2 (step 5) deals with the analysis method. Category 3 (steps 6–8) studies sample preparation, while category 4 (steps 9–11) investigates the consumed solvents and reagents. Eventually, category 5 (steps 12–15) manifests the influence of instrumentation on the ecosystem. The proposed method's greenness was assessed by checking the impact for each step under the guidance of GAPI [31], imparting it either a green, yellow or red colour, describing it to be green, moderately green, or hazardous analytical tool respectively [31]. Table 5 summarizes the GAPI index of the suggested assay, where its greenness is demonstrated. Meanwhile, figure 8 depicts GAPI pictograms illustrating the significant greenness of this work.

**Table 5.** GAPI parameters description of the proposed method.

category	step	description	green	yellow	red
sample collection	1	at line		√	
sample preservation	2	none	√		
sample transport	3	none	√		
sample storage	4	none	√		
method type	5	direct	√		
extraction	6	none	√		
used solvents	7	water		√	
sample treatment	8	none	√		
solvent volume	9	less than 10 ml	√		
health hazard	10	non-irritant, nontoxic	√		
safety hazard	11	no safety hazard	√		
consumed energy	12	less than 0.1 W per sample	√		
occupational hazard	13	none	√		
waste volume	14	1–10 ml		√	
waste treatment	15	biodegradable		√	

## 4. Conclusion

The proposed method succeeded to quantitatively analyse each of CIP and LEV simultaneously with their photolytic products obtained at different pH values by applying FDSFS as an analysis tool. The suggested assay proved to be simple, sensitive, selective and cost-effective, which permits its widespread application in laboratories, without the need for experienced analysts. The photodegradation kinetics of both analytes were studied and found to be comparable to former reports. Moreover, the obtained kinetic data were depicted to postulate the environmental photofate of the studied FQN and to predict the antibacterial activity of the resultant photolytic products. Furthermore, the proposed assay was found to follow the basic principles of green chemistry as indicated by the applied GAPI. The pronounced advantages of the suggested analytical tool make it a convenient alternative to other methods encountered in environmental analysis.

## Data Availability

Data are available from the Dryad Digital Repository under title: Fluorescence spectra presenting kinetic photodegradation of ciprofloxacin and levofloxacin: https://doi.org/10.5061/dryad.cvdncjt71 [46].
